# Rapid Prototyping of a Nanoparticle Concentrator Using a Hydrogel Molding Method

**DOI:** 10.3390/polym13071069

**Published:** 2021-03-29

**Authors:** Hirotada Hirama, Ryutaro Otahara, Katsuo Mogi, Masanori Hayase, Toru Torii, Harutaka Mekaru

**Affiliations:** 1 Human Augmentation Research Center, National Institute of Advanced Industrial Science and Technology, Chiba 277-0882, Japan; h-mekaru@aist.go.jp; 2 Faculty of Science and Technology, Tokyo University of Science, Chiba 278-8510, Japan; chikuzenni2826@gmail.com (R.O.); mhayase@rs.tus.ac.jp (M.H.); 3 Molecular Profiling Research Center for Drug Discovery, National Institute of Advanced Industrial Science and Technology, Tokyo 135-0064, Japan; mogi.k@aist.go.jp; 4 Future Center Initiative, The University of Tokyo, Chiba 277-0871, Japan; torii@edu.k.u-tokyo.ac.jp

**Keywords:** ion concentration polarization, concentration, microfluidics

## Abstract

Nanoparticle (NP) concentration is crucial for liquid biopsies and analysis, and various NP concentrators (NPCs) have been developed. Methods using ion concentration polarization (ICP), an electrochemical phenomenon based on NPCs consisting of microchannels, have attracted attention because samples can be non-invasively concentrated using devices with simple structures. The fabrication of such NPCs is limited by the need for lithography, requiring special equipment and time. To overcome this, we reported a rapid prototyping method for NPCs by extending the previously developed hydrogel molding method, a microchannel fabrication method using hydrogel as a mold. With this, we fabricated NPCs with both straight and branched channels, typical NPC configurations. The generation of ICP was verified, and an NP concentration test was performed using dispersions of negatively and positively charged NPs. In the straight-channel NPC, negatively and positively charged NPs were concentrated >50-fold and >25-fold the original concentration, respectively. To our knowledge, this is the first report of NP concentration via ICP in a straight-channel NPC. Using a branched-channel NPC, maximum concentration rates of 2.0-fold and 1.7-fold were obtained with negatively and positively charged NPs, respectively, similar to those obtained with NPCs fabricated through conventional lithography. This rapid prototyping method is expected to promote the development of NPCs for liquid biopsy and analysis.

## 1. Introduction

The concentration of nanoparticles (NPs) is crucial in the field of liquid biopsy and analysis [1,2,3], and various NP concentrators (NPCs) have been developed [3,4,5]. NPCs improve the detection limits of biomarkers (e.g., NPs such as exosomes) for cancer and dementia [5] and the throughput of post-concentration analysis and processing [3]. In recent years, NPCs using ion concentration polarization (ICP), which is an electrochemical phenomenon, have been developed [5]. These devices mainly consist of a microchannel with a built-in selective ion permeation structure (e.g., ion exchange membrane), can concentrate samples non-invasively [6], and have a simple structure because no internal electrodes are required [7,8]. ICP is an electrochemical ion transport phenomenon that occurs near the selective ion transmission structure owing to the application of an electric field [9,10,11,12,13,14]. Due to the generation of ICP in the microchannel, an electrically neutral region, specifically the ion depletion zone (IDZ), which is locally deficient in ions, is formed. The IDZ can eliminate charged substances [15,16]. By utilizing this property, an ICP-based NPC can retain and concentrate the charged substance on the upstream side of the IDZ. Typical types of ICP-based NPCs include those with a straight channel [17] and those with a branched channel [15]. NPCs with a straight channel are used for on-chip batch analysis (where a concentrated sample is retained in a channel) and can concentrate NPs at relatively high concentration rates, whereas it is difficult to collect concentrated samples (which are retained in a channel) outside of the device [18]. NPCs with branched channels are used to continuously concentrate charged substances without the retention of the concentrated sample in a channel. Whereas the concentration rate is lower than that with the straight-channel NPC, the concentrated sample can be recovered from the device [18].

NPCs consisting of microchannels have been fabricated through a variety of rapid prototyping techniques for microchannel fabrication, using transparent, easy-to-process polydimethylsiloxane (PDMS) with low cytotoxicity [19] as a device material [3,4,5]. Photo- and soft-lithography [20] is a conventional rapid prototyping technique that is time-consuming and requires special equipment such as mask aligners, clean rooms, and plasma irradiators [21]. To further facilitate the development of NPCs for liquid biopsy and analysis, an NPC rapid prototyping method that enables us to design, prototype, and evaluate NPCs without special equipment is required.

We have previously reported a method of fabricating PDMS-based microchannels using a hydrogel as a mold (that is, the hydrogel molding method (HGM)) [22,23,24,25]. The HGM process does not require the special equipment that is used for photo- and soft-lithography. A hydrogel mold used in the HGM can be easily fabricated by casting a wire-shaped hydrogel from capillary tubes (e.g., a glass capillary and a polytetrafluoroethylene tube). HGM enables the simple and rapid preparation of two-dimensional and three-dimensional PDMS-based microchannels with various cross-sectional shapes (e.g., circles, squares, triangles). Although HGM could impart various properties to microchannels by embedding different materials in PDMS together with a hydrogel mold, HGM has not been applied to the production of such microchannels.

In this study, to overcome the existing issue (i.e., the fabrication of NPCs is limited by the need for lithography, requiring special equipment and time), we developed a rapid prototyping method for NPCs consisting of microchannels using the HGM. In the proposed method, a commercially available ion-exchange membrane (Nafion) was embedded in the device together with the hydrogel mold, and special equipment used for conventional photo- and soft-lithography techniques was not required. Here, two typical types of devices (an NCP with a straight channel and an NCP with a branched channel) were prepared. First, the fabrication conditions (i.e., temperature and Nafion membrane width) were optimized. Subsequently, the generation of ICP in the prepared NCPs was verified using a fluorescent dye. Finally, the concentration performance of the NCPs was evaluated using NPs.

## 2. Materials and Methods

### 2.1. Preparation of Sample Dispersion

To verify the ICP generated in an NPC, we prepared a 300 µM aqueous solution of uranine (fluorescent dye, Fujifilm Wako Pure Chemical Industries, Ltd., Osaka, Japan), which can visualize IDZ [21]. To investigate the device characteristics for NP concentration, two different dispersions, positively charged NPs (designed with amino groups on the particle surface; mean diameter: 227.8 ± 25.5 nm, measured by dynamic light scattering; Micromer^®^-redF, Micromod Partikeltechnologie GmbH, Rostock, Germany) and negatively charged NPs (designed with carboxy groups on the particle surface; mean diameter: 273.7 ± 32.2 nm, measured by dynamic light scattering; Micromer^®^-redF, Micromod Partikeltechnologie GmbH, Germany), were prepared at 6.0 × 10^10^ particles/mL.

### 2.2. Device Fabrication Using a Hydrogel Molding Method

**Figure 1:**
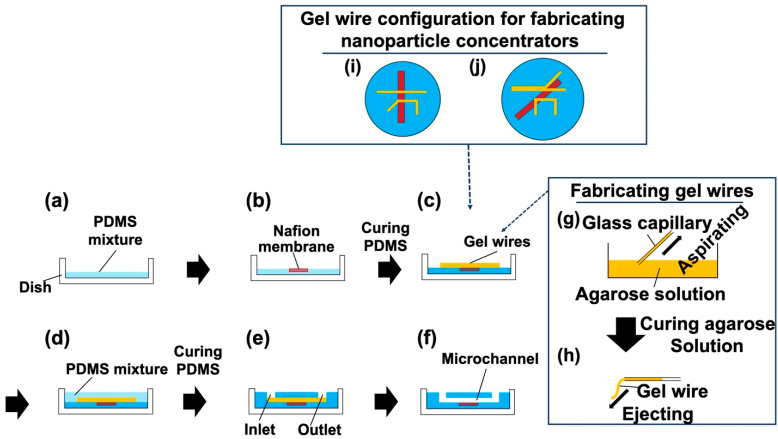
Nanoparticle concentrator fabrication procedures using a hydrogel molding method: (**a**) the polydimethylsiloxane (PDMS) mixture was poured into a dish; (**b**) the Nafion membrane was placed onto the PDMS, and the PDMS was cured; (**c**) gel wires were placed on the cured PDMS; (**d**) the PDMS mixture was used as filling on the gel wires, and curing the PDMS was performed at 53 °C for 2 h; (**e**) openings of inlet and outlet holes, achieved with a biopsy punch; (**f**) the gel wires were washed out with hot water; (**g**) agarose solution was used for filling into a glass capillary, and this was cured at 4 °C for 10 min; (**h**) the gel wire was ejected from the glass capillary; (**i**) gel wire configuration for fabricating a nanoparticle concentrator with a straight channel; and (**j**) gel wire configuration for fabricating a nanoparticle concentrator with a branched channel.

Figure 1 shows the procedure for preparing an NPC using the HGM. First, the bottom part of the NPC was prepared as follows. A polystyrene dish (60 mm in diameter, Corning, St Louis, MO, USA) was poured with 2.5 g of a polydimethylsiloxane (PDMS; SILPOT 184, Dow Toray, Japan) mixture (base agent:curing agent = 10:1 weight ratio; Figure 1a). Then, a Nafion membrane (NRE 212, thickness: 50.8 μm, Sigma Aldrich, St Louis, MO, USA; length 3 cm) was placed on the uncured PDMS and heated to cure (Figure 1b). Preliminary studies have shown that the heating temperature and the width of the Nafion membrane affect the wrinkle and deflection formation of the Nafion membrane, and the wrinkles and deflections can inhibit the occurrence of ICP. To investigate the morphology of the bottom part of the fabricated NPC, NPCs were fabricated at different heating temperatures (25, 35, 45, 55, 65, and 75 °C) using Nafion membranes with different widths (2, 4, 6, and 8 mm). The heating times were set to 12 h at 25 and 35 °C, 3 h at 45 °C, and 2 h at 55 °C and 65 °C. Subsequently, the gel wires used as molds were prepared as follows. A solution of 16% (*v*/*v*) glycerol (Fujifilm Wako Pure Chemical Industries, Ltd., Osaka, Japan) aqueous solution, 3% (*w*/*v*) agarose (Agarose L, Fujifilm Wako Pure Chemical Industries, Ltd., Osaka, Japan) aqueous solution, and 0.01% (*w*/*v*) blue food dye (Brilliant Blue FCF, Fujifilm Wako Pure Chemical Industries, Ltd., Japan) aqueous solution were prepared. The agarose solution was aspirated in a rectangular glass capillary (cross-section: 0.5 mm × 0.5 mm, Takao Manufacturing CO., LTD., Kyoto, Japan) and cooled at 4 °C for 10 min to cure. Gel wires (cured agarose) were ejected from the glass capillary by applying positive pressure. Subsequently, the gel wires were placed on the bottom part of the NPC, as previously described herein (Figure 1c). In this study, two different configurations of gel wires (i.e., straight configuration for fabricating an NPC with a straight channel and branched configuration for an NPC with a branched channel) were prepared (Figure 1i,j). The PDMS mixture (10 g) was poured onto the gel wires and heated at 53 °C for 2 h to cure (Figure 1d). In HGM, by curing the PDMS mixture poured onto uncured PDMS, a gap-free structure was formed without a bonding process [22,23,24,25]. Two types of biopsy punches (tip diameter of 2 mm for the outlet and 3 mm for the inlet, Kai Industries Co., Ltd., Gifu, Japan) were used to punch holes in the cured PDMS (Figure 1e). Hot water was poured through the holes to dissolve and remove the gel wires (Figure 1f).

### 2.3. Generation of Ion Concentration Polarization

**Figure 2:**
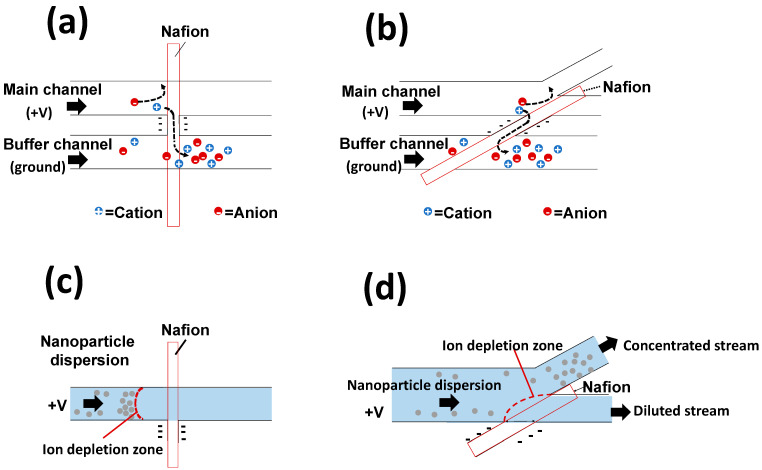
Schematics of the ion concentration polarization (ICP)-driven ion transfer and nanoparticle concentration in microchannels. Ion movement in (**a**) a nanoparticle concentrator with a straight channel and (**b**) a nanoparticle concentrator with a branched channel. Nanoparticle concentration in (**c**) a nanoparticle concentrator with a straight channel and (**d**) a nanoparticle concentrator with a branched channel.

**Figure 3:**
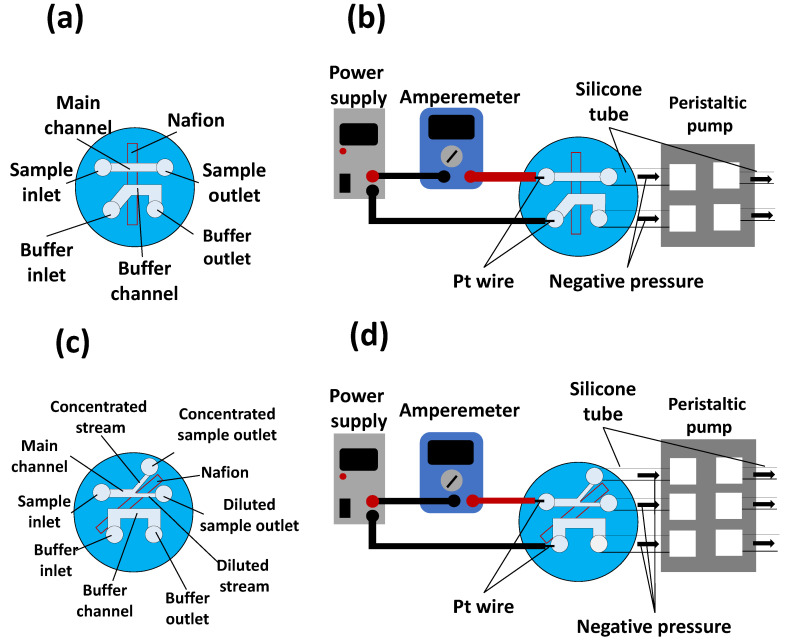
Device systems for nanoparticle concentration: (**a**) schematics of a nanoparticle concentrator with a straight channel; (**b**) setup for a nanoparticle concentrator with a straight channel; (**c**) schematics of a nanoparticle concentrator with a branched channel; and (**d**) setup for a nanoparticle concentrator with a branched channel.

To concentrate the sample dispersions (i.e., fluorescent dye and NPs), we generated ICP in an NPC. Here, we prepared two types of NPCs (an NPC with a straight channel and an NPC with a branched channel; Figure 2) using the HGM previously described herein. To generate ICP, we applied voltage with a power supply (P4K-80M, Matsusada Precision Inc., Osaka, Japan) and platinum wires (diameter: 0.5 mm, length: 10 cm, Kennis Ltd., Osaka, Japan). When a voltage is applied to the NPC, cations are transported from the main channel to the buffer channel via the Nafion membrane (Figure 2a,b). This ion transport reduces the concentration of cations in the main channel. In the region where the concentration of cations is low, the relative excess of anions repel each other to maintain electrical neutrality. As a result, IDZ, a region depleted of cations and anions, is formed [5]. As IDZ acts as a partition wall that blocks the charged substances [5,15], an NPC with a straight channel can concentrate NPs charged upstream of IDZ (Figure 2c). An NPC with a branched channel can concentrate and recover charged NPs by removing the electrically neutral medium (i.e., water). Figure 3 shows device systems for nanoparticle concentration. An NPC was connected to containers filled with a sample dispersion and buffer liquid (pure water) through a silicone tube (inner diameter: 1 mm, outer diameter: 2 mm) and was connected to a peristaltic pump (MTIC05-01, ICOMES LAB Co., Ltd., Iwate, Japan) through a silicone tube. To uniformly introduce a sample dispersion and buffer liquid to each channel in the NPC, negative pressure was applied to the outlet using the peristaltic pump. Sample dispersion and pure water were introduced into the main channel and buffer channel, respectively.

### 2.4. Characterization

Scanning electron microscopy (VHX-D510, KEYENCE Co., Ltd., Osaka, Japan) was used to observe the morphology of the prepared NPCs. To observe the behavior of the sample in an NPC, a fluorescence microscope (BZ-X710, KEYENCE Co., Ltd., Japan) was used. ImageJ software (National Institutes of Health, Bethesda, MD, USA) was used for the image analysis. As an NPC with a branched channel has a structure for which a concentrated sample stream can be separated from the main sample stream, a concentrated NP dispersion was collected from the concentrated sample outlet. A spectrophotometer (NANO DROP ONE^C^, Thermo Fisher Scientific, Waltham, MA, USA) was used to measure the NP concentrations in the collected NP dispersions. An ammeter (DT4282, HIOKI E.E. Corporation, Nagano, Japan) was used to measure the current in the NPC.

## 3. Results and Discussions

### 3.1. Fabricated Nanoparticle Concentrator

**Figure 4:**
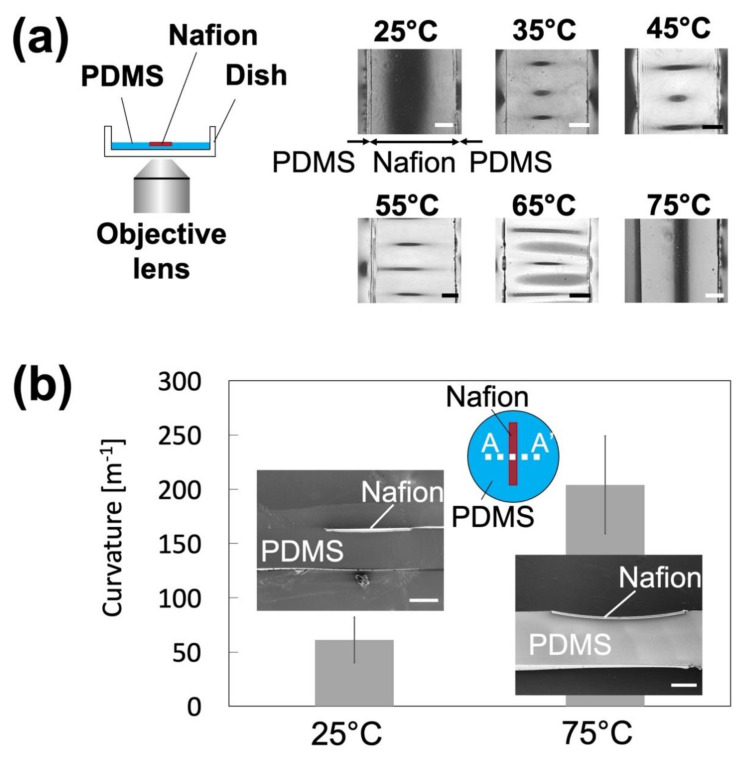
Effect of the heating temperature and Nafion membrane width on the shape of the Nafion membrane on the bottom part of the nanoparticle concentrator: (**a**) Nafion membrane on polydimethylsiloxane (PDMS), prepared by heating. The scale bars represent 500 μm. The vertical and horizontal black lines on the membrane are the curvature and wrinkles, respectively; (**b**) relationship between heating temperature and curvature. Inserts are scanning electron microscopy images of the A–A’ cross-section. The scale bars represent 500 μm (*n* = 5 samples).

**Figure 5:**
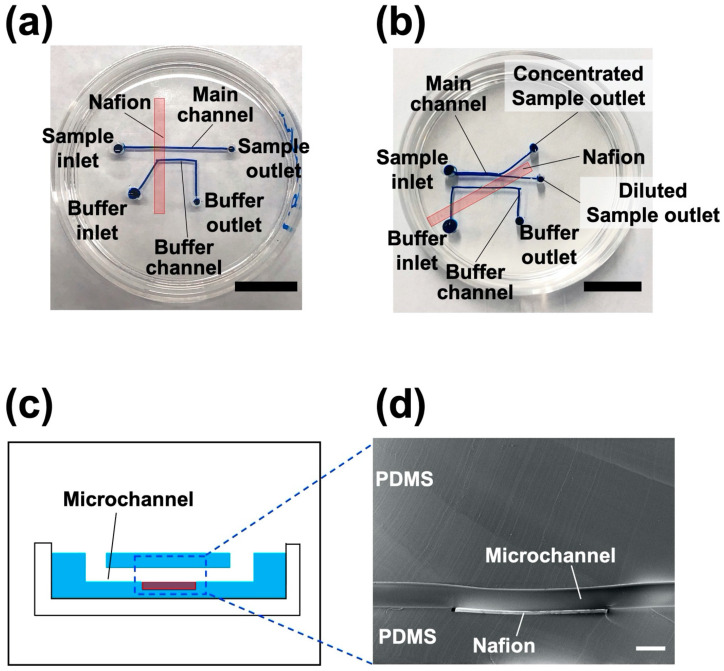
Fabricated nanoparticle concentrators: (**a**) a nanoparticle concentrator with a straight channel. The scale bar represents 2 cm; (**b**) a nanoparticle concentrator with a branched channel. The scale bar represents 2 cm; (**c**) a schematic diagram; and (**d**) a scanning electron microscopy image of the cross-section of a microchannel with a Nafion membrane. The scale represents 500 µm. PDMS, polydimethylsiloxane.

First, to form a uniform Nafion membrane on the bottom part of an NPC, the effects of the heating temperature and Nafion membrane width were investigated. No wrinkles were observed on the Nafion membrane (width: 2 mm) at 25 and 75 °C, whereas wrinkled structures were formed on the Nafion membrane at 35–65 °C (Figure 4a). The curvature of the Nafion membrane was smaller at 25 °C than at 75 °C (Figure 4b). These results suggest that (1) the PDMS heated above 35 °C expands thermally and then returns at room temperature, causing PDMS and membrane contraction; and (2) at a higher heating temperature (75 °C), the membrane was deformed owing to the evaporation of water in the membrane [26]. From these results, the heating temperature was set to 25 °C in subsequent experiments. Then, at a constant heating temperature (25 °C), changes in the curvature of the Nafion membranes were examined using membranes of different widths. The curvatures of the obtained membranes were almost constant regardless of the membrane width (Figure S1). This shows that in this fabrication method, the membrane width [17,27], which is one of the parameters related to the behavior of ICP, can be uniformly formed in the range of 2–8 mm at a heating temperature of 25 °C. In the following experiments, an NPC incorporating a 2 mm-wide Nafion membrane was used. Figure 5 shows fabricated nanoparticle concentrators. In this study, we successfully produced an NPC with a straight channel with a branched channel in which a Nafion membrane was embedded in the bottom part of the NPC without wrinkles.

### 3.2. A Nanoparticle Concentrator with a Straight Channel

#### 3.2.1. Device Validation for Ion Concentration Polarization

**Figure 6:**
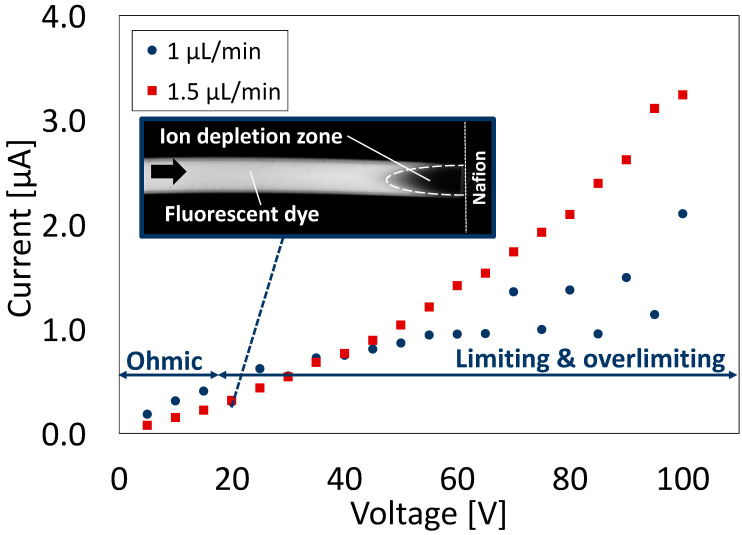
Ion concentration polarization visualization and current–voltage (I–V) characteristics of a nanoparticle concentrator with a straight channel at different flow rates (*n* = 60). Fluorescent dye aqueous solution was used for the sample dispersion. Inserts are fluorescence microscope images showing the behavior of the fluorescent dye in the channel (flow rate: 1 μL/min).

To verify that ICP is generated in a prepared NPC with a straight channel, ICP visualization and current–voltage (I–V) characterization were performed using an aqueous fluorescent dye solution, as performed in a previous study [21] (Figure 6). The ICP behavior was investigated at two different flow rates. At a relatively low flow rate (1 μL/min), the formation of an IDZ (which shows ICP generation) was observed above 20 V by increasing the applied voltage. The current value showed a three-step increase with respect to the applied voltage, shown as follows. Up to 20 V, the current tended to increase as the applied voltage increased. When the applied voltage exceeded 20 V, the current increased slightly. As the applied voltage increased, the increase in current tended to increase again. Similarly, previous studies [28,29,30] have reported that ICPs generated in ion-selective permeable membranes incorporated into microchannels exhibit a three-step current response depending on the applied voltage. In the voltage range below the threshold, the I–V curve follows Ohm’s law. Therefore, this voltage range is called the Ohmic region. In the voltage range above the threshold, ICP occurs, and the current value is almost constant or drops (i.e., the resistance increases). This voltage range is called the limiting region. At applied voltages higher than the limiting region, electroosmotic instability (EOI) occurs, resulting in the creation of vortices near the ion exchange membrane. This vortex disturbs the IDZ, and the current value simultaneously increases again. This voltage range is referred to as the overlimiting region. In the current study, the behavior of an NPC with a straight channel was consistent with that reported in previous studies [28,29,30]. Additionally, when the applied voltage exceeded 65 V, the current value greatly increased with fluctuations up and down as the voltage increased. This behavior was not observed in previous studies [29]. The greater the height of the microchannel, the more likely vortexes will occur due to EOI, and as a result, vortexes cause the instability of IDZ formation [31]. As a microchannel fabricated by HGM has a deeper structure than that in previous studies [29,31], vortexes might have been more likely to occur, and the IDZ destabilized by the vortex could have caused fluctuations in the current value.

However, at a relatively higher flow rate (1.5 µL/min), no IDZ (which shows no ICP formation) was observed in the applied voltage range of this study. The current value increased as the applied voltage increased. This voltage range is in the Ohmic region. Under this flow rate, a stepwise change in the increase in the current value according to the applied voltage did not occur. In a higher flow environment, the expansion (formation) of the IDZ can be constricted by supplying more ions from the upstream compared to that in a lower flow environment. This might have caused the constant resistance in the channel (i.e., the Ohmic region).

#### 3.2.2. Nanoparticle Concentration

**Figure 7:**
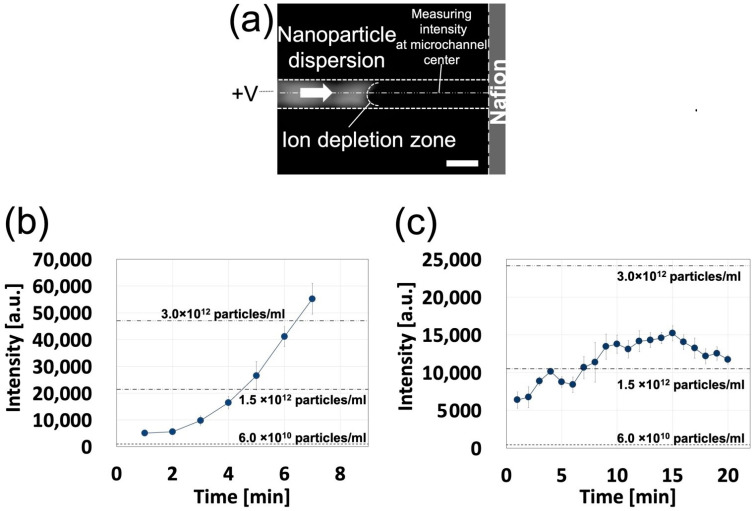
Nanoparticle concentration in a nanoparticle concentrator with a straight channel at a constant flow rate (2 μL/min) and with a constant applied voltage (80 V); (**a**) a fluorescent microscope image. The scale bar represents 500 µm (negatively charged nanoparticles (NPs)); (**b**) time course of fluorescence intensity in negatively charged nanoparticles within the channel (*n* = 5); and (**c**) the time course of fluorescence intensity in positively charged nanoparticles within the channel (*n* = 5).

Using an NP dispersion, the behavior of the ICP-based NP concentration in a prepared NPC with a straight channel was investigated (Figure 7). In this study, two different NPs (negatively and positively charged NPs) were introduced into the NPC, and fluorescence microscope images were acquired every minute for 20 min after the voltage was applied. By analyzing the acquired fluorescence microscope images, a line profile of the fluorescence intensity at the center of the microchannel (position: 250 μm from the wall surface of the flow path) was obtained. The time course of the maximum fluorescence intensity on the line profile was plotted (Figure 7b,c). According to a previous study [18], the ICP-based NP concentration in an NPC with a straight channel is more stable at lower flow rates and higher applied voltages. Based on this, the conditions of the lowest flow rate and the highest voltage were determined from the range of ICP occurrence in a preliminary study (Figure S2); for negatively charged NPs, the applied voltage was set to 80 V and the flow rate was set to 2 µL/min, whereas for positively charged NPs, the applied voltage was set to 90 V and the flow rate was set to 2 µL/min.

In both negatively charged NPs and positively charged NPs, the fluorescence intensity tended to increase over time. For negatively charged NPs, the NP concentration reached more than 50 times the original concentration 7 min after the voltage was applied, and the fluorescence intensity (concentration) reached the camera sensitivity limit (saturation) 8 min after the voltage was applied. For positively charged NPs, the fluorescence intensity peaked (more than 25 times the original concentration) 15 min after the voltage was applied. Subsequently, the fluorescence intensity gradually decreased. This decrease in fluorescence intensity was due to the fact that the NPs concentrated by ICP gradually spread downstream from the membrane. This diffusion (leakage) of NPs downstream might have been caused by the retention of NPs at a concentration higher than that which IDZ could block. In addition, the fluorescence intensity (concentration) of negatively charged NPs was higher than that of positively charged NPs (more than 3.0 × 10^12^ particles/mL). It has been reported that positively charged nanomaterials (proteins) introduced into PDMS-based NPCs at low flow rates are non-specifically adsorbed on the channel surface, and the concentration rate is lower than that of negatively charged proteins [15]. Similarly, in this study, positively charged NPs were non-specifically adsorbed on the channel surface, and the concentration rate was lower than that of negatively charged NPs. To our knowledge, this is the first report of the condensation of NPs by ICP in a concentrator with a straight channel (note: the previous work [18] has concentrated molecules rather than NPs).

### 3.3. A Nanoparticle Concentrator with a Branched Channel

#### 3.3.1. Device Validation for Ion Concentration Polarization

**Figure 8:**
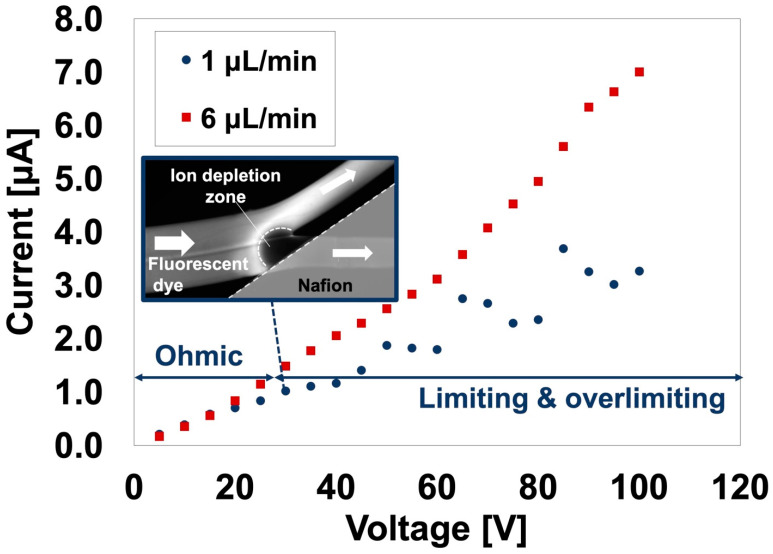
Ion concentration polarization visualization and current-voltage (I–V) characteristics of a nanoparticle concentrator with a branched channel at different flow rates. A fluorescent dye aqueous solution was used as sample dispersion (*n* = 60). Inserts are fluorescence microscope images showing the behavior of the fluorescent dye in the channel (applied voltage: 30 V).

To verify that ICP is generated in a prepared NPC with a branched channel, ICP visualization and I–V characterizations were performed using an aqueous fluorescent dye solution (Figure 8). Similar to that with an NPC with a straight channel, the ICP behavior was investigated at two different flow rates. At a relatively low flow rate (1 μL/min), the formation of an IDZ was observed above 30 V by increasing the applied voltage. As with an NPC with a straight channel, the NPC with a branched channel exhibited a three-step current response, and the current value increased with fluctuations going up and down as the voltage increased. However, at a relatively higher flow rate (6 µL/min), no IDZ was observed in the applied voltage range in this study. Additionally, as the applied voltage increased, the current increased without pulsation. These behaviors were the same as those in an NPC with a straight channel. However, in an NCP with a branched channel, the minimum applied voltage forming the IDZ was larger than that in an NCP with a straight channel. This might be because the difference in the channel structure caused a difference in the electrical resistance in the channel. This should be investigated further in the future.

#### 3.3.2. Nanoparticle Concentration

**Figure 9:**
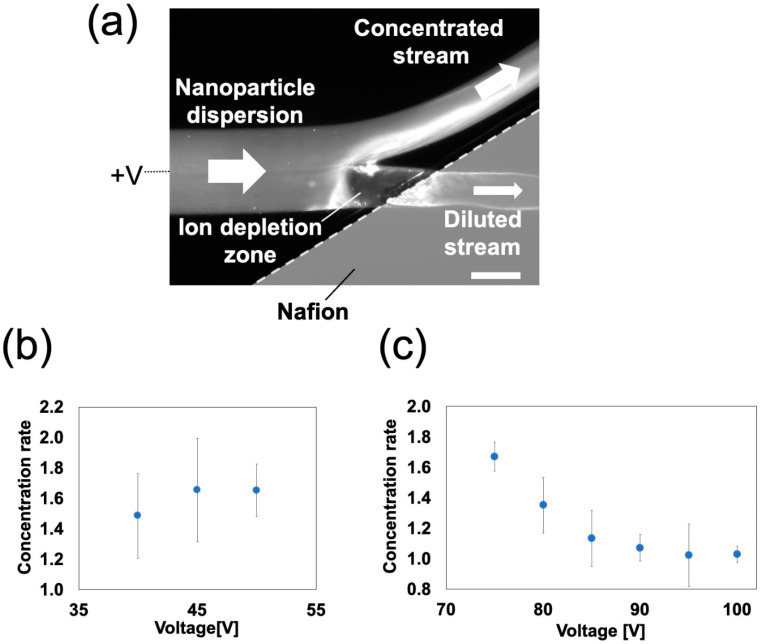
Nanoparticle (NP) concentration in a nanoparticle concentrator with a branched channel: (**a**) a fluorescent microscope image (flow rate: 6 µL/min, applied voltage: 100 V, negatively charged NPs). The scale bar represents 300 µm. Relationship between the applied voltage and concentration rate in (**b**) negatively charged nanoparticles and (**c**) positively charged nanoparticles (flow rate: 2 µL/min; *n* = 5).

The behavior of the ICP-based concentration was investigated in an NPC with a branched channel using NP dispersion (Figure 9). Here, a voltage was applied after introducing two different nanoparticles, as in an NPC with a straight channel. Thirty minutes after the voltage was applied, the treated NP dispersion was removed from the NPC, and the NP concentration was measured using a spectrophotometer. The concentration was determined from the obtained concentrations. The concentration rate was defined as follows:

(1) $$Concentration\;rate=\frac{Concentration\;of\;processed\;nanoparticle\;dispersion}{Concentration\;of\;original\;nanoparticle\;dispersion}$$

Similar to those in an NPC with a straight channel, to achieve a stable ICP-based NP concentration, the conditions of the lowest flow rate and highest voltage were determined from the range of ICP occurrences with a preliminary study (Figure S3); for negatively charged NPs, the applied voltage was set to 40–50 V and the flow rate was set to 2 µL/min, and for positively charged NPs, the applied voltage was set to 75–100 V and the flow rate was set to 2 µL/min.

For negatively charged NPs, the average concentration rates were increased 1.5–1.7-fold (Figure 9b). There was no clear correlation between the concentration rate and the applied voltage. The concentration of negatively charged NPs due to stable ICP formation was observed upstream of the channel junction. The concentrated NPs flowed into the concentrated stream. However, for positively charged NPs, the average concentration rates were 1.0–1.7-fold higher (Figure 9c). As the applied voltage increased, the concentration rate decreased. Owing to the stable ICP formation, the IDZ expanded towards the concentrated stream as the applied voltage increased. This blocked the flow of concentrated NPs that were about to flow into the concentrated stream. The concentrated NPs that could not flow into the concentrated stream stagnated on the upstream side of the IDZ. As reported in a previous study [5], the IDZ tends to expand as the applied voltage increases. Similarly, in the NPCs produced in this study, the IDZ expanded as the applied voltage increased. This increased the proportion of NPs stagnating on the upstream side of the IDZ, resulting in a decrease in the concentration rate. The maximum concentration rates for negatively and positively charged NPs were 2.0 times (Figure S4) and 1.7 times higher (Figure 9c), respectively. In an NPC with a branched channel prepared by existing fabrication techniques (photo- and soft lithography) [5], the concentration rates (measured from the fluorescence intensity in the NPC) for negatively and positively charged NPs were 1.8 and 1.3 times higher, respectively. These results show that an NPC with a branched channel fabricated by HGM has similar concentration properties to that fabricated via the existing method. Under the ideal condition that all nanoparticles flow into the concentrated stream (i.e., no leakage into the diluted stream), the concentration rate (*r*) can be expressed by the following equation [15]:

(2) $$r=\frac{Q_{1}+Q_{2}}{Q_{1}}$$

where *Q*_1_ and *Q*_2_ represent flow rates in the concentrated stream and the diluted stream, respectively. Here, because *Q*_1_ and *Q*_2_ were set to the same value, it was expected that *r* would be 2. This shows that the maximum concentration rate would be two-fold higher using Equation (2). However, in this experiment and the aforementioned previous study, *r* did not reach 2. These results suggest that both of these devices caused nanoparticles to leak into the diluted stream.

In this study, an NPC with a constant angle of the branched channel orientation was used and the angle effect on the concentration rate was not considered, as in a previous study [15]. However, according to the previous study, *r* in an NPC with a branched channel can be expressed as follows.

(3) $$r=1+\frac{R_{1}}{R_{2}}$$

where *R*_1_ and *R*_2_ represent hydrodynamic resistances in the concentrated stream and the diluted stream, respectively. As the angle of the branched channel orientation can affect the pressure drop (which affects *R*_1_), the angle might affect the concentration rate.

## 4. Conclusions

Using a hydrogel as a mold, we successfully fabricated an NPC with a straight channel and an NPC with a branched channel without the need for special equipment used for conventional photo- and soft-lithography techniques. In both of these NPCs, the generation of ICP allowed the NPs to be concentrated. In an NPC with a straight channel, the fluorescence intensity in the channel tended to increase over time for both negatively charged and positively charged NPs. In addition, negatively charged NPs reached a concentration of more than 50 times the original concentration, and positively charged NPs reached a concentration of more than 25 times the original concentration. To our knowledge, this is the first report of the enrichment of NPs by ICP in a concentrator with a straight channel. In an NPC with a branched channel, the concentrated NPs could be removed from the NPC, and the maximum concentration rates for negatively and positively charged NPs were 2.0 and 1.7 times higher, respectively. These concentration rates were similar to those of an NPC with a branched channel prepared via existing photo- and soft-lithography. The rapid prototyping method proposed in this study is expected to facilitate the development of NPCs for liquid biopsy and microfluidics-based analysis, including the concentration and measurements targeting exosomes and viruses.

## Data Availability

The data presented in this study are available on request from the corresponding author.

## Supplementary Materials

The following are available online at https://www.mdpi.com/article/10.3390/polym13071069/s1, Figure S1: Relationship between the Nafion membrane width and the curvature at a constant heating temperature (25 °C), Figure S2: The range of ion concentration polarization (ICP) occurrence in a nanoparticle concentrator (NPC) with a straight channel, Figure S3: The range of ion concentration polarization (ICP) occurrence in a nanoparticle concentrator with a branched channel, Figure S4: Concentration of negatively charged nanoparticles in a nanoparticle concentrator with a branched channel at a constant flow rate.
