# Supplementary material for: Rapid Prototyping of a Nanoparticle Concentrator Using a Hydrogel Molding Method
Source: Polymers (Basel). 2021 Mar 29;13(7):1069. doi: 10.3390/polym13071069 (PMC8037731; doi:10.3390/polym13071069)
Supplement: Supplementary file 1 [file polymers-13-01069-s001.pdf]

polymers

MDPI

Supplementary Materials

Rapid prototyping of a nanoparticle concentrator using a hydrogel molding method

Hirotada Hirama [1,*,†], Ryutaro Otahara [2,†], Katsuo Mogi [3], Masanori Hayase [2], Toru Torii [4], and Harutaka Mekaru [1]

1 Human Augmentation Research Center, National Institute of Advanced Industrial Science and Technology, Chiba 277-0882, Japan; h-mekaru@aist.go.jp (H.M.)
2 Faculty of Science and Technology, Tokyo University of Science, Chiba 278-8510, Japan; chikuzenni2826@gmail.com (R.O.); mhayase@rs.tus.ac.jp (M.H.)
3 Molecular Profiling Research Center for Drug Discovery, National Institute of Advanced Industrial Science and Technology, Tokyo 135-0064, Japan; mogi.k@aist.go.jp (K.M.)
4 Future Center Initiative, The University of Tokyo, Chiba 277-0871, Japan; torii@edu.k.u-tokyo.ac.jp (T.T.)
* Correspondence: h.hirama@aist.go.jp; Tel.: +81-29-861-3065
† These authors contributed equally to this work

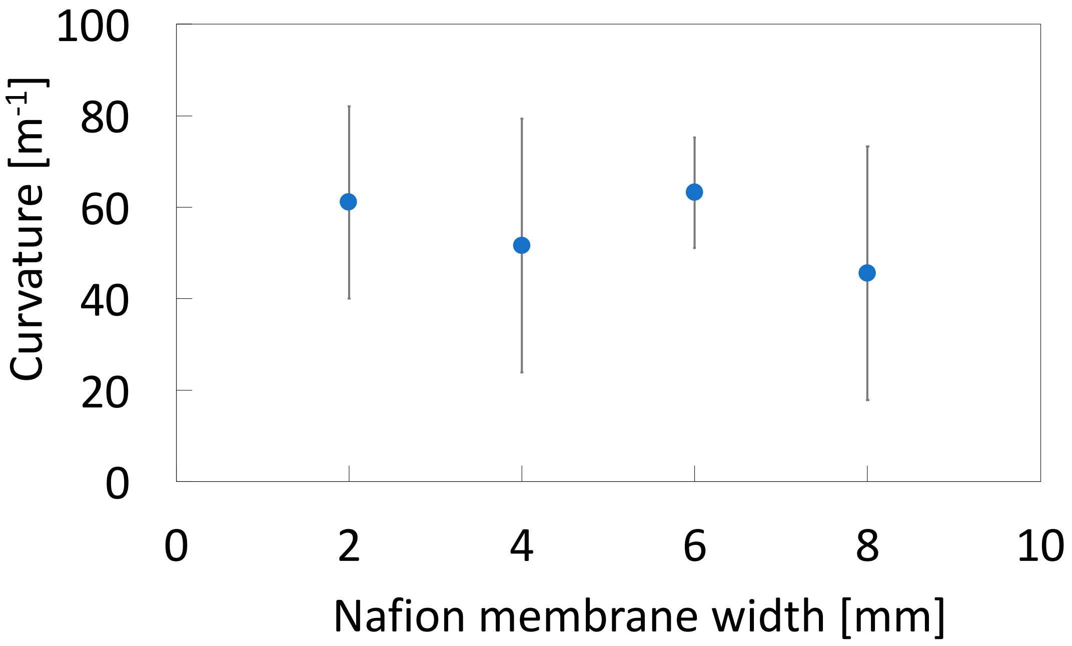

Figure S1. Relationship between Nafion membrane width and curvature at a constant heating temperature (25°C) (n = 5).

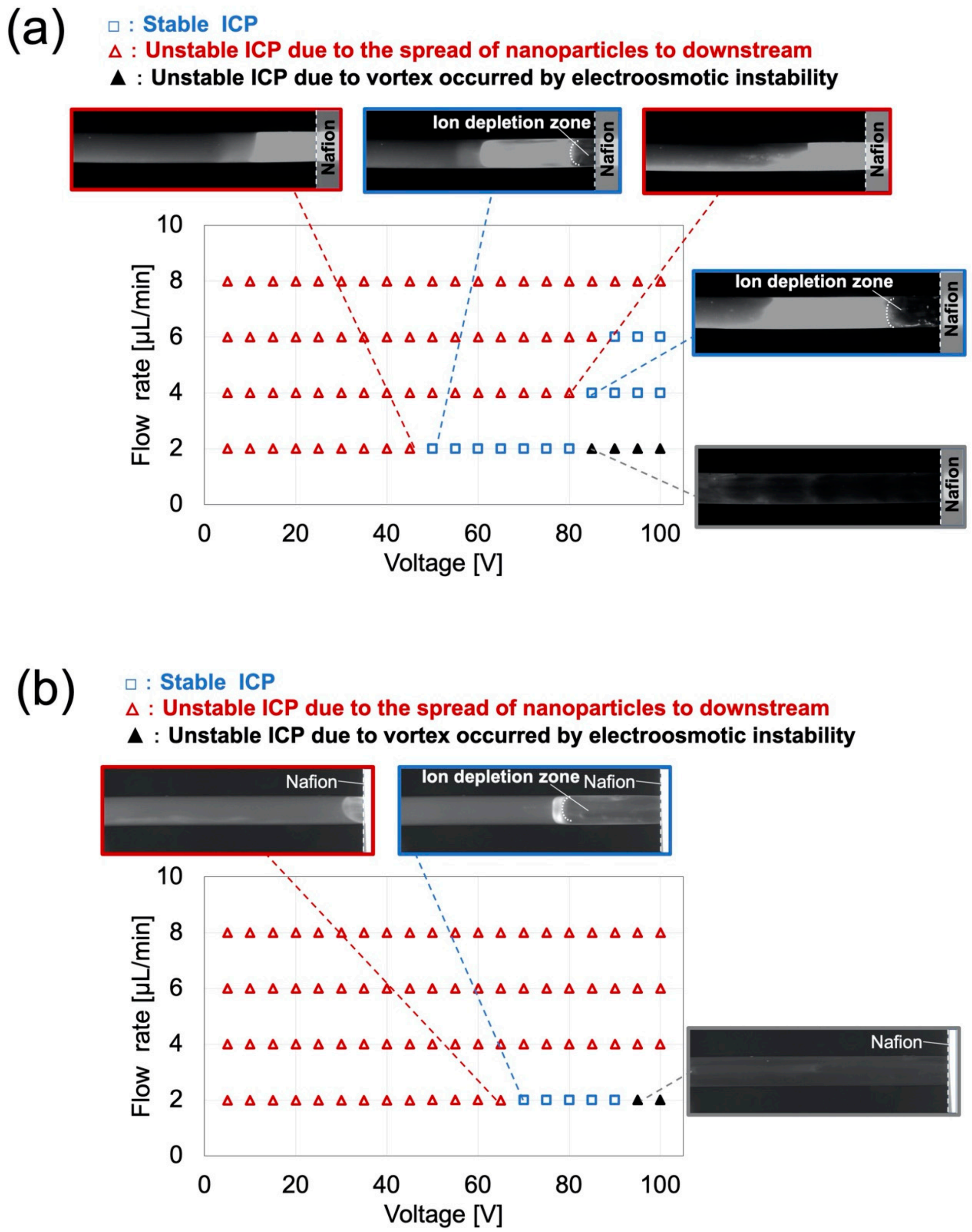

Figure S2. The range of ion concentration polarization (ICP) occurrence in a nanoparticle concentrator (NPC) with a straight channel. Nanoparticle (NP) dispersions were used as sample dispersions. We obserbed the NP behavior in NPC; i.e., the ion depletion zone (IDZ) formation on the upstream side of the Nafion membrane for a certain period (50 s) after the voltage was applied. The conditions under which IDZ was retained were defined as □, and the conditions under which IDZ could not be retained were defined as △ and ▲. Under the condition of △, the nanoparticles were affected by the flow and diffused downstream. Under the condition of ▲, the IDZ was violently disturbed by the vortex generated near a Nafion membrane. This pushed back the nanoparticles to the upstream side, resulting in movement of the nanoparticles out of the camera view. (a) Negatively charged nanoparticles. (b) Positively charged nanoparticles.

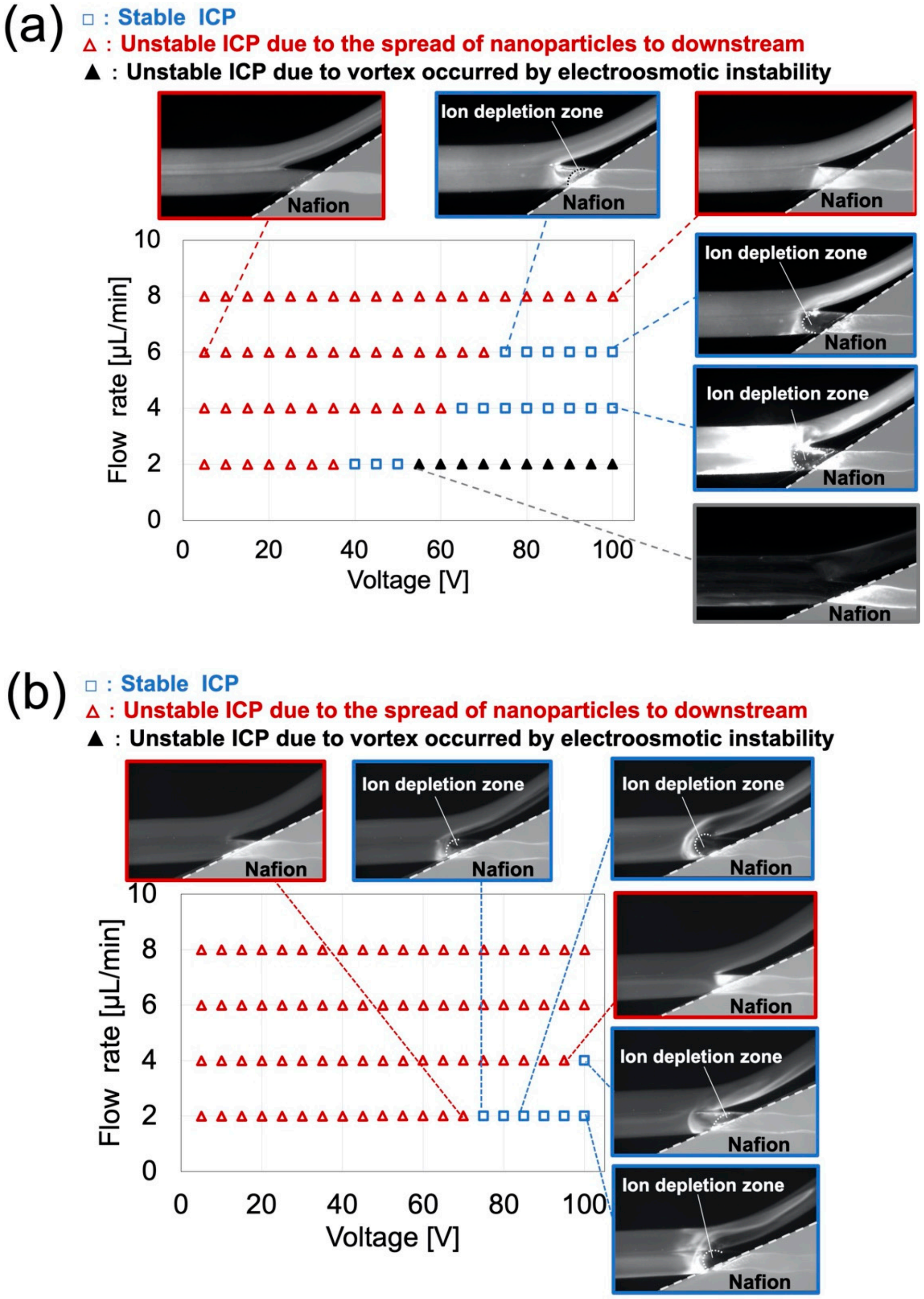

Figure S3. The range of ion concentration polarization (ICP) occurrence in a nanoparticle concentrator with a branched channel. Nanoparticle dispersions were used as sample dispersions. We obserbed the NP behavior in NPC; i.e., the ion depletion zone (IDZ) formation on the upstream side of the Nafion membrane for a certain period (50 s) after the voltage was applied. The conditions under which IDZ was retained were defined as □, and the conditions under which IDZ could not be retained were defined as △ and ▲. Under the condition of △, the nanoparticles were affected by the flow and diffused downstream. Under the condition of ▲, the IDZ was violently disturbed by the vortex generated near a Nafion membrane. This pushed back the nanoparticles to the upstream side, resulting in movement of the nanoparticles out of the camera view. (a) Negatively charged nanoparticles. (b) Positively charged nanoparticles.

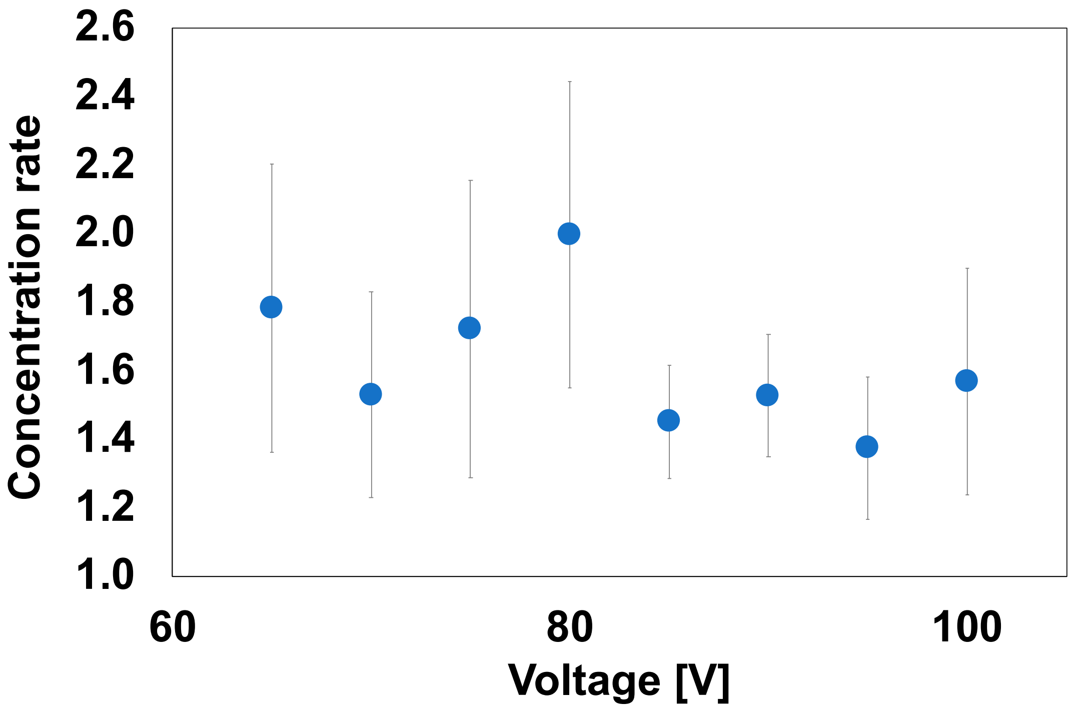

Figure S4. Concentration of negatively charged nanoparticles in a nanoparticle concentrator with a branched channel at constant flow rate (4 µL/min; n = 5).
